# Effect of carbon on whole-biofilm metabolic response to high doses of streptomycin

**DOI:** 10.3389/fmicb.2015.00953

**Published:** 2015-09-11

**Authors:** Lindsay M. D. Jackson, Otini Kroukamp, Gideon M. Wolfaardt

**Affiliations:** ^1^Department of Chemistry and Biology, Ryerson University, TorontoON, Canada; ^2^Department of Microbiology, Stellenbosch UniversityStellenbosch, South Africa

**Keywords:** biofilm, antibiotic, metabolism, high doses, recovery

## Abstract

Biofilms typically exist as complex communities comprising multiple species with the ability to adapt to a variety of harsh conditions. In clinical settings, antibiotic treatments based on planktonic susceptibility tests are often ineffective against biofilm infections. Using a CO_2_ evolution measurement system we delineated the real-time metabolic response in continuous flow biofilms to streptomycin doses much greater than their planktonic susceptibilities. Stable biofilms from a multispecies culture (containing mainly *Pseudomonas aeruginosa* and *Stenotrophomonas maltophilia*), Gram-negative environmental isolates, and biofilms formed by pure culture *P. aeruginosa* strains PAO1 and PAO1 ΔMexXY (minimum planktonic inhibitory concentrations between 1.5 and 3.5 mg/l), were exposed in separate experiments to 4000 mg/l streptomycin for 4 h after which growth medium resumed. In complex medium, early steady state multispecies biofilms were susceptible to streptomycin exposure, inferred by a cessation of CO_2_ production. However, multispecies biofilms survived high dose exposures when there was extra carbon in the antibiotic medium, or when they were grown in defined citrate medium. The environmental isolates and PAO1 biofilms showed similar metabolic profiles in response to streptomycin; ceasing CO_2_ production after initial exposure, with CO_2_ levels dropping toward baseline levels prior to recovery back to steady state levels, while subsequent antibiotic exposure elicited increased CO_2_ output. Monitoring biofilm metabolic response in real-time allowed exploration of conditions resulting in vulnerability after antibiotic exposure compared to the resistance displayed following subsequent exposures.

## Introduction

Most biofilms are formed by multispecies microbial communities (Lindsay and von Holy, 2006). Though biofilms have many positive roles in nature, industry, and for human health, they pose a pronounced risk to immunocompromised individuals. More than 65% of bacterial infections in humans are caused due to bacteria forming biofilms and 10–20% of nosocomial infections are caused by the formation of biofilms on medical devices (Nithya et al., 2010). Once a biofilm has formed in a chronic wound it is difficult for the host’s immune system to eradicate (Fux et al., 2005), and 1000s of deaths and billions of dollars in medical costs are incurred each year from such infections (Dowd et al., 2008). A variety of bacterial species are often isolated from biofilm infections, with two common pathogens associated with nosocomial infections being *Stenotrophomonas maltophilia* and *Pseudomonas aeruginosa* (Pedersen et al., 1992; Dowd et al., 2008; Tseng et al., 2009; Nithya et al., 2010). *Stenotrophomonas* and *Pseudomonas* species are associated with a number of illnesses and have been co-isolated in several wound types and infections (Tseng et al., 2009; Araoka et al., 2010).

With a large proportion of infections caused by bacterial biofilms, many efforts have been made to understand antibiotic resistance, as antibiotics remain a main treatment of bacterial infections (Martin and Ernst, 2003; Hanlon, 2007). Earlier reports indicated that bacterial biofilms can resist concentrations of antibiotics up to 1000 times greater than their planktonic counterparts (Nickel et al., 1985; Gristina et al., 1987; Mah and O’Toole, 2001; Nithya et al., 2010) though there are studies that point out that this is a false impression (Spoering and Lewis, 2001). Biofilm antibiotic resistance has been linked to various conditions and behaviors specific to the biofilm environment, such as reduced antibiotic penetration due to adsorption to matrix components or degradation by extracellular enzymes (Stewart and Costerton, 2001), slower growth (Ashby et al., 1994), expression of eﬄux pumps (Brooun et al., 2000), expression and/or increased local concentrations of antibiotic-modifying or -degrading enzymes (Giwercman et al., 1991; O’Toole et al., 2000), alterations in antibiotic targets (O’Toole et al., 2000), nutrient limitation (Dorrer and Teuber, 1977; Nguyen et al., 2011), adaptive stress responses (Nguyen et al., 2011), and the presence of persister cells (Spoering and Lewis, 2001; Stewart, 2002).

Although having antibiotic resistance genes is important for bacterial resistance, the physiological state of bacteria can greatly affect their susceptibility since antibiotics traditionally target various forms of macromolecular synthesis (Hancock, 1981; Eng et al., 1991; Hooper, 2001; Cotsonas King and Wu, 2009). Thus, it can be expected that nutrients and energy sources affect bacterial susceptibility to antimicrobials. Earlier work with *P. aeruginosa* demonstrated that the minimum inhibitory concentration (MIC) of colistin and polymyxin B was dependent on carbon source in which the bacteria were cultivated (Conrad, 1979). Another study found that carbon sources entering in upper glycolysis (such as glucose and mannitol) potentiate aminoglycoside killing of bacterial persister cells as compared to carbon sources that entered lower glycolysis (such as succinate or citrate; Allison et al., 2011). Furthermore, eﬄux pump mediated resistance in clinically relevant Gram-negative bacteria can be reduced by substituting the culture medium with non-phosphotransferase system sugars that cause changes in protein concentration in the periplasmic space, which limit eﬄux pump activity (Villagra, 2012).

The preceding studies described how various carbon sources affect antibiotic susceptibility by investigating how variations in substrate can affect bacterial growth, antibiotic uptake, and cellular composition in planktonic systems. Since bacterial biofilms are highly resistant to antibiotics, we explored how whole-biofilm metabolism and antibiotic susceptibility were affected when grown in various carbon sources and when additional carbon was added during antibiotic exposure. The antibiotic streptomycin was used, which is an aminoglycoside that targets protein synthesis, and can disrupt the integrity of the bacterial outer membrane (Taber et al., 1987). Both streptomycin uptake and eﬄux are energy-dependent processes (Taber et al., 1987; Webber and Piddock, 2003) and certain antibiotic modifying enzymes may require energy, which could all be affected by nutritional status. Thus, energy dependent processes and the net flux of the antibiotic could influence the survival. Finding the mechanisms of antibiotic resistance was not the primary objective of this study, though an eﬄux pump mutant was subjected to the same conditions to determine if a similar response would be observed. Through careful monitoring of whole-biofilm metabolic response to high doses of antibiotics in various nutrient conditions we hope to demonstrate important aspects of biofilm behavior that can be further explored with gene, protein, or cellular based methods.

To test how changes in medium and carbon content affect biofilm susceptibility, the CO_2_ evolution measurement system (CEMS) (Kroukamp and Wolfaardt, 2009) was used. The goal was to delineate the real-time metabolic response of whole biofilms of a *S. maltophilia*–*P. aeruginosa* containing multispecies culture, as well as several environmental isolates when exposed to high concentrations of streptomycin in complex and defined growth media with varying carbon concentrations. Genomic analyses of the starting cultures obtained from freezer stocks and a bench culture revealed the presence of *S. maltophilia*, despite the frequent subculturing of the bench culture on agar plates. Further metagenomic analysis was performed on biofilm and biofilm eﬄuent (before and after antibiotic exposure), which showed the presence of *S. maltophilia* in the cultures as well. Streptomycin was applied at concentrations a 100 to a 1000 times greater than the planktonic MIC, in accordance with the frequent reference to these high values in the literature (e.g., Mah and O’Toole, 2001). We observed increases in metabolism upon antibiotic addition. This led us to hypothesize that the addition of carbon during antibiotic exposures may aid in biofilm recovery from high dose streptomycin exposures, in contrast to the notion that biofilms survive high concentration antibiotic exposures due to inactivity. Keeping in mind that biofilms are spatially highly heterogeneous, our aim was to elucidate trends in whole-biofilm metabolic behavior.

## Materials and Methods

### Bacterial Cultures

Three Gram-negative environmental strains (*Enterobacter asburiae, Enterobacter* sp., and *P. putida)* isolated from a washroom sink drain (Ghadakapour et al., 2014), and a multispecies culture containing *P. aeruginosa* and *S. maltophilia* were used in this study to compare metabolic behavior among Gram-negative isolates exposed to streptomycin. The bacteria in the multispecies biofilm containing *P. aeruginosa* and *S. maltophilia* were determined by full genome sequencing and metagenomic analysis and the environmental strains were identified by 16S rRNA. Further testing was done on single species biofilms of *P. aeruginosa* strains PAO1 and PAO1 ΔMexXY (Fraud and Poole, 2011) to observe how eﬄux pump activity affects response to high dose streptomycin exposures. Continuous flow systems (see below) were inoculated from freezer stocks as well as bench cultures repeatedly sub-cultured on agar plates. All pre-cultures were grown in either 3 g/l tryptic soy broth (TSB, EMD Chemicals, Billerica, MA, USA; which contains 1.4 mM glucose) or in a defined growth medium with final concentrations of 1.51 mM (NH_4_)_2_SO_4_, 3.37 mM Na_2_HPO_4_, 2.20 mM KH_2_PO_4_, 179 mM NaCl, 0.1 mM MgCl_2_⋅H_2_O, 0.01 mM CaCl_2_⋅2H_2_O, and 0.001 mM FeCl_3_ with 5 mM glucose or sodium citrate (Clark and Maaløe, 1967) at 37°C, while agitated [300 rotations per minute (rpm)].

### Antibiotic Minimum Inhibitory Concentration (MIC)

A stock solution of streptomycin sulfate (Bio Basic Inc. Markham, ON, CA) with a final concentration of 10,000 mg/l was prepared following a protocol described earlier (Andrews, 2001). The MIC of streptomycin for the multispecies culture containing *P. aeruginosa* and *S. maltophilia* strains, *E. asburiae, Enterobacter* sp., and *P. putida* were determined in 3 g/l TSB (Ghadakapour et al., 2014). In addition, biofilm eﬄuent MIC was obtained for multispecies biofilms inoculated from bench cultures (see biofilm eﬄuent collection described below). MIC’s were determined at 25°C for all isolates, as well as at 37°C for the multispecies culture. Although MIC’s are generally performed at 37°C, testing at 25°C was performed in order to measure planktonic antibiotic susceptibility at the same temperature used for biofilm antibiotic testing. Approximately 10^7^ cells (100 μl from a 10^8^ cells/ml suspension) of overnight culture were added to the 5 ml antibiotic dilutions and incubated overnight with shaking at 300 rpm, at either 37°C or 25°C for 18–20 h. The antibiotic concentrations tested ranged from 1.75 to 200 mg/l streptomycin and each dilution tested was performed in triplicate. A positive control (10^7^ cells from the overnight culture added to sterile 3 g/l TSB without antibiotic) and a negative control (sterile 3 g/l TSB medium without any inoculum) were prepared for each experiment. The MIC was determined at the concentration of antibiotic that resulted in no growth of the culture (as determined by no turbidity or cloudiness seen in the culture).

### Biofilm Development

**FIGURE 1:**
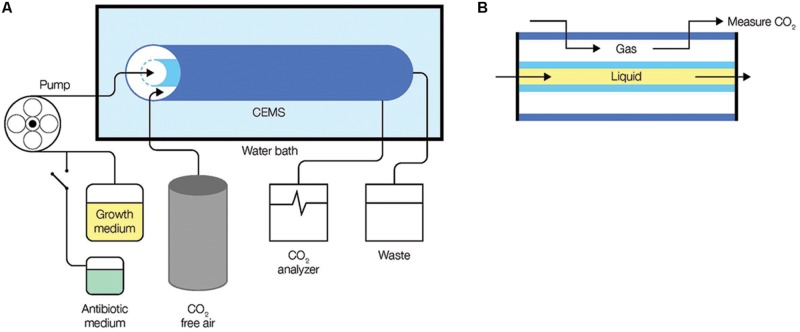
**Monitoring of biofilm metabolism through the capture of CO_2_ production in real-time. (A)** The CEMS system was set-up with media being fed into a gas-permeable inner silicone tube in the CEMS using a peristaltic pump. CO_2_ released by biofilms in the inner tube cross the silicon wall into the annular space confined by a gas-impermeable outer tygon tube, from where it is collected and transferred by CO_2_-free sweeper gas to a CO_2_ analyzer. **(B)** A cross-section of the CEMS showing flow of growth medium (liquid) and gas, separated by a gas-permeable silicone.

A carbon dioxide evolution measurement system (CEMS) was used to grow biofilms. In this system, a continuous flow of growth medium is fed into inner silicone tubing where the biofilm grows. The silicone tubing is permeable to gas enabling the CO_2_ produced by the biofilm to be collected by a CO_2_-free sweeper gas and measured in real time through a CO_2_ analyzer (Kroukamp et al., 2010). Growth medium, with and without added antibiotic, was fed into the CEMS at a flow rate of 15 ml/h (hydraulic retention time of 8 min) using a peristaltic pump (**Figure 1**). Planktonic cells were being washed away faster than they can multiply within the tube because the dilution rate exceeds planktonic bacterial specific growth rates by at least 10 times. The CEMS apparatus was immersed in a water bath kept at 25°C. Biofilms were fed continuously with 0.3 g/l TSB medium until they reached metabolic levels that corresponded with late exponential phase, early steady state, or late steady state levels prior to the aminoglycoside exposures. For inoculation, 1 ml of the respective pre-cultures was introduced into the CEMS without flow for up to 60 min before flow of media was resumed. Batch-grown pre-cultures were always cultivated in the same complex (TSB) or defined medium as the biofilm under investigation.

For the purpose of the biofilm experiments, low and high TSB concentrations were considered to be 0.3 g/l (1% of manufacturer’s recommended concentration containing 0.14 mM glucose) and 3 g/l (1.4 mM glucose), respectively. In the case of the defined medium, high concentrations of carbon were considered to be at 1 mM for citrate and glucose or 2 mM for pyruvate, while citrate used at a concentration of 0.14 mM was considered a low carbon medium.

### Antibiotic Exposure

The CO_2_ profile produced by biofilms grown in the CEMS was used to determine when the biofilms had reached a metabolically-stable state between 24–48 h after inoculation (early steady state biofilms). Streptomycin sulfate (Biobasic Inc., Markham, ON, CA) was added directly to sterile medium and the biofilms were exposed to streptomycin concentrations of 4000 mg/l up to 12000 mg/l for 4 h; the physiological half-life of streptomycin in human plasma is 2–4 h, thus a 4-h exposure time was chosen (Clarke, 1986). All of the antibiotic exposures were performed at a minimum in duplicates, mostly numerous replicates except for the antibiotic exposures on the two environmental isolates *E. asburiae* and *Enterobacter* sp.

### Viable Cell Counts in Biofilm Eﬄuent

Eﬄuent samples were collected to determine the viability and numbers of planktonic cells being released from biofilms before, during, and after antibiotic exposures. Biofilm eﬄuent samples collected from steady state biofilms fed with antibiotic-free growth medium were serially diluted before plating. Biofilm eﬄuent samples collected during antibiotic treatment were washed twice via centrifugation at 12000 × *g* for 150 s and re-suspended in 0.9% saline solution to reduce the presence of residual antibiotic before plating on 3 g/l TSA plates and incubated at 37°C.

### pH Controls

The pH of the media used in this study ranged from 6.71 to 7.09 and the pH of the antibiotic containing media ranged between 6.14 and 6.36. The ratio of dissolved CO_2_ to bicarbonate ions increases at lower pH. Since it is the dissolved CO_2_ that crosses the silicone tube wall, it could be argued that sudden increases in measured CO_2_ upon a decrease in pH could be attributed to this pH (and dissolved CO_2_ ratio) shift alone. However, for any pH where the bicarbonate ion to dissolved CO_2_ ratio may increase, the entire reactor volume would still be replaced within 8 min, therefore any measured CO_2_ changes due to pH-induced dissolved CO_2_ ratio would be transient.

## Results

### Antibiotic Susceptibility of Young Multispecies Biofilms Grown in 0.3 g/l TSB Medium

**FIGURE 2:**
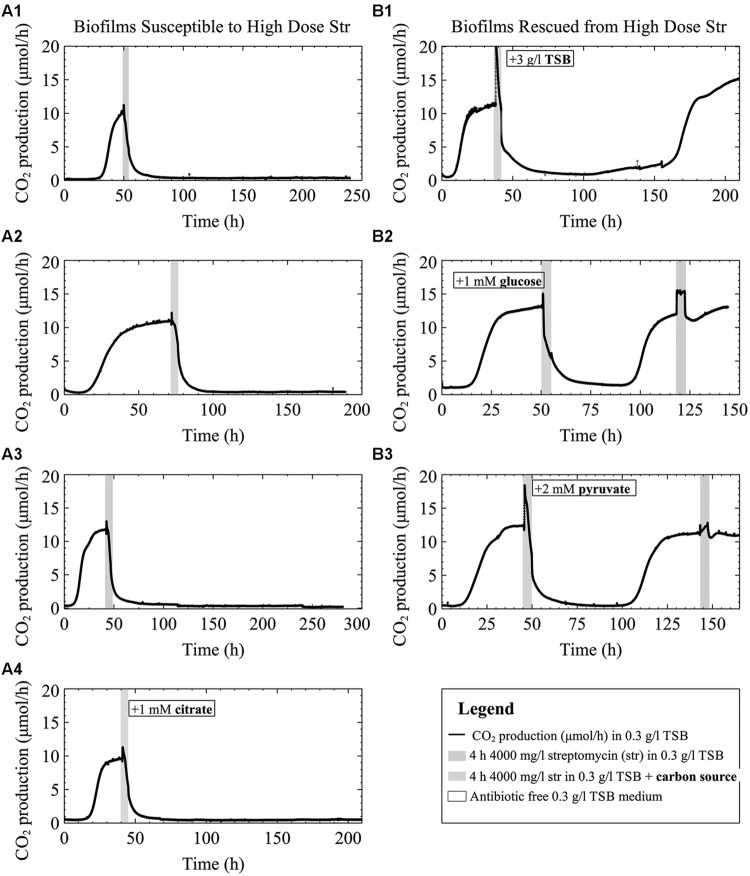
**CO_2_ production (μmol/h) of multispecies biofilms grown on 0.3 g/l TSB medium exposed to 4000 mg/l streptomycin with and without the addition of carbon to the antibiotic medium.** Column **(A)** shows late exponential phase **(A1)** and early steady state biofilms **(A2–A4)** exposed to streptomycin. The biofilms were exposed to streptomycin in either 0.3 g/l TSB medium **(A1–A3)** or 0.3 g/l TSB medium with the addition of 1 mM citrate to the 0.3 g/l TSB antibiotic medium **(A4)**. Column **(B)** shows the metabolic response of biofilms that recovered following the high dose streptomycin exposures from the addition of either 3 g/l TSB **(B1)**, 1 mM glucose **(B2)**, or 2 mM pyruvate **(B3)** to the antibiotic medium.

To test the notion that biofilms can withstand antibiotic concentrations 10 to a 1000 times their planktonic MIC, multispecies-biofilms (inoculum planktonic MIC between 1.5–3.5 mg/l) were exposed to 4000 mg/l of streptomycin. As previously observed (Kroukamp et al., 2010), biofilms formed by *Pseudomonas* strains may have notably different lengths in lag phase that will cause the biofilm to stabilize CO_2_ production at different times depending on the inoculum. Thus we considered biofilm growth stage as criterion for when to introduce the antibiotic – a factor deserving to be considered in biofilm research. Biofilms grown until just before the onset of steady state and early steady state CO_2_ production were susceptible to 4000 mg/l of streptomycin (**Figures 2A1–A3** and Figure SM1 in Supplementary Material). Even when provided with antibiotic-free growth medium for up to 7 days following exposure, the biofilms did not recover.

### Effect of Added Carbon During Antibiotic Exposure on Young Biofilms Grown in 0.3 g/l TSB Medium (Containing 0.14 mM Glucose)

Biofilms exposed to high doses of streptomycin (**Figures 2A1–A3**) demonstrated a brief spike in CO_2_ production upon addition of the antibiotic medium. This innate tendency to increase metabolism at the onset of streptomycin addition led us to hypothesize that added energy during an antibiotic exposure, especially for early steady state biofilms, would decrease susceptibility of biofilms grown in low concentration (0.3 g/l) TSB. We attempted to demonstrate this (similar to biofilms shown in **Figures 2A2,A3**) by providing additional carbon during the antibiotic exposure. Indeed, when biofilms grown in 0.3 g/l TSB were exposed to 4000 mg/l streptomycin dissolved in high concentration (3 g/l) TSB, there was an immediate spike in metabolism, followed by a drop in CO_2_ production to near baseline levels and subsequent recovery over a period of more than 100 h to return to steady state (**Figure 2B1**). Similarly, we were able to rescue the biofilms grown in 0.3 g/l TSB when exposed to streptomycin with the addition of 1 mM glucose or 2 mM pyruvate (**Figures 2B2,B3**). Unlike glucose or pyruvate addition, addition of 1 mM citrate to the antibiotic medium did not result in the rescue of the biofilm (**Figure 2A4**). When comparing the graphs in column A to the graphs in column B there are a few observations worth mentioning: in column B, when additional carbon sources are added to the antibiotic media (**Figure 2**) the initial increases in CO_2_ output are greater than those in column A when no carbon is added along with the antibiotic, or when citrate is added to the antibiotic medium. Furthermore, the CO_2_ outputs of the biofilms in column B (**Figures 2B1,B2**) do not drop completely to base line levels prior to biofilm recovery toward steady state CO_2_ output.

### Effect of Additional Carbon During Antibiotic Exposures on Biofilms Grown in Low Carbon (0.14 mM) Defined Medium

Bacteria behave differently when provided with various nutrient sources, in either complex or minimal media (Rojo, 2010). In addition, *P. aeruginosa* prefers organic acids (as carbon sources) to glucose (Ng and Dawes, 1967). Thus, we were interested to test if *P. aeruginosa* biofilms grown in a defined medium with citrate as the sole carbon source [at a concentration equal to that of the glucose (0.14 mM) in the 0.3 g/l TSB medium] would show similar sensitivity to the antibiotic as when grown in 0.3 g/l TSB medium.

**FIGURE 3:**
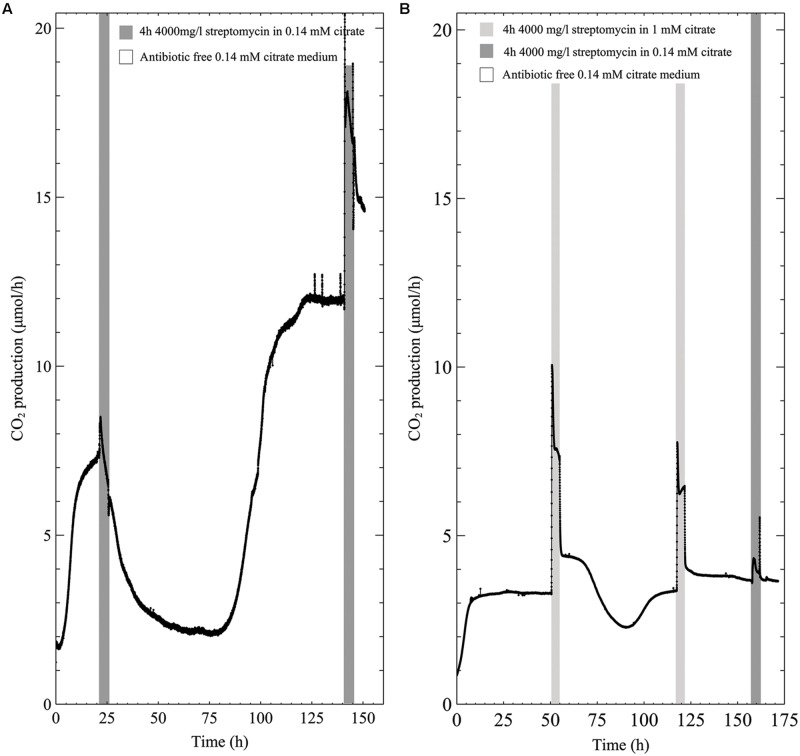
**CO_2_ production (μmol/h) of multispecies biofilms grown in 0.14 mM citrate defined medium and exposed to 4000 mg/l streptomycin for 4-h intervals; **(A)** 0.14 mM citrate medium **(B)** 0.14 mM citrate growth medium with the addition of 1 mM citrate during the first and second exposure, and a third antibiotic exposure in 0.14 mM citrate medium**.

The metabolic profile of early steady state multispecies biofilms grown in low concentration (0.14 mM) citrate medium showed a slight increase in CO_2_ production upon antibiotic exposure before CO_2_ levels decreased toward baseline levels (**Figure 3A**). However, unlike the susceptibility to the antibiotic in 0.3 g/l TSB, the biofilms were able to recover from the 4000 mg/l streptomycin exposure even in the case of early steady state biofilm. Furthermore, a second exposure to the same concentration of streptomycin resulted in an increase in biofilm metabolism that was maintained throughout the 4-h exposure. When the biofilms grown in low citrate (0.14 mM) concentration medium were exposed to antibiotic medium supplemented with 1 mM citrate, CO_2_ production rapidly increased and remained above steady state values for the duration of the antibiotic exposure. Following the resumption of antibiotic-free growth medium after the first antibiotic exposure, there was a drop in biofilm metabolism below steady state levels that rapidly recovered, though subsequent exposures returned to steady state metabolism immediately following the removal of the antibiotic medium (**Figure 3B**).

### Biofilm Metabolic Response while Switching Carbon Sources with and without Antibiotic Present

**FIGURE 4:**
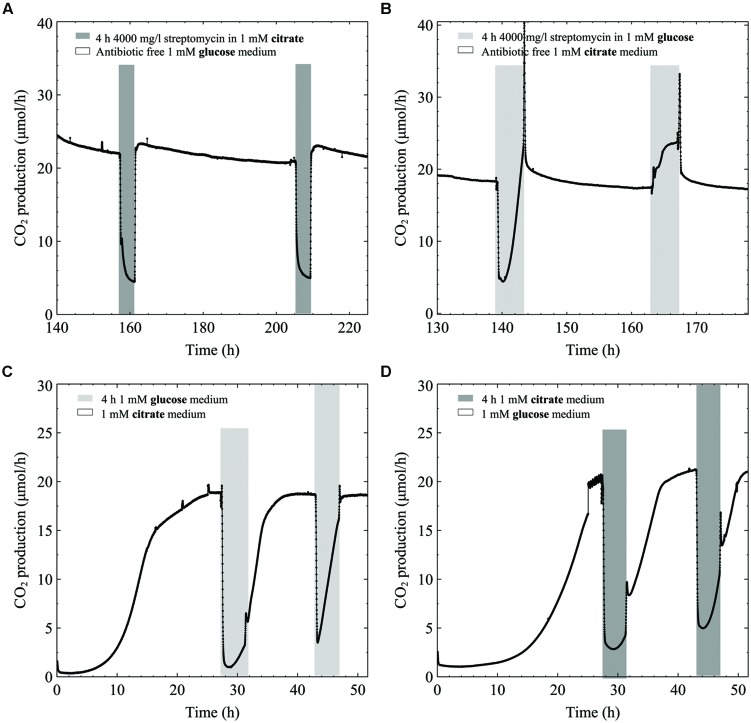
**CO_2_ production (μmol/h) of multispecies biofilms grown in defined medium: **(A)** 1 mM glucose as the carbon source exposed two times to 4000 mg/l streptomycin in 1 mM citrate, **(B)** 1 mM citrate exposed two times to 4000 mg/l streptomycin in 1 mM glucose, **(C)** 1 mM citrate grown biofilms exposed to 1 mM antibiotic free glucose and **(D)** 1 mM glucose grown biofilms exposed to 1 mM antibiotic free citrate**.

While biofilms grown from the start in defined medium with citrate as carbon source were resistant to high streptomycin concentrations, added citrate could not rescue TSB grown biofilms from streptomycin. To explore citrate’s inability to rescue biofilms exposed to streptomycin in TSB (glucose containing) growth medium (**Figure 2A4**), we aimed to delineate biofilm metabolic changes when switching from citrate to glucose medium with and without antibiotics present. When biofilms were grown in a defined medium containing 1 mM glucose and exposed to antibiotic in 1 mM citrate, the metabolism dropped by ∼80% at the end of the 4-h exposure, with rapid recovery once switched back to antibiotic free 1 mM glucose medium (**Figure 4A**). When biofilms were grown in 1 mM citrate medium and exposed to the antibiotic in 1 mM glucose medium there was a similar initial drop in metabolism when the carbon source was switched. Conversely, the metabolism recovered before the end of exposure, while further exposures resulted in an increase in metabolism for the 4-h duration (**Figure 4B**). To demonstrate the effects of switching the carbon source on biofilm metabolism without any antibiotic present we switched from either 1 mM citrate or glucose media to a 4 h 1 mM glucose or citrate exposure. The CO_2_ production of biofilms shuts down instantaneously when switching from either glucose medium to citrate and vice versa. Without antibiotic present both glucose and citrate biofilms started to increase their CO_2_ output levels again during mid exposure. Interestingly, the return to steady state CO_2_ output took longer when antibiotic was not present in the media (**Figures 4C,D**).

### Potential Role of Eﬄux Pump

**FIGURE 5:**
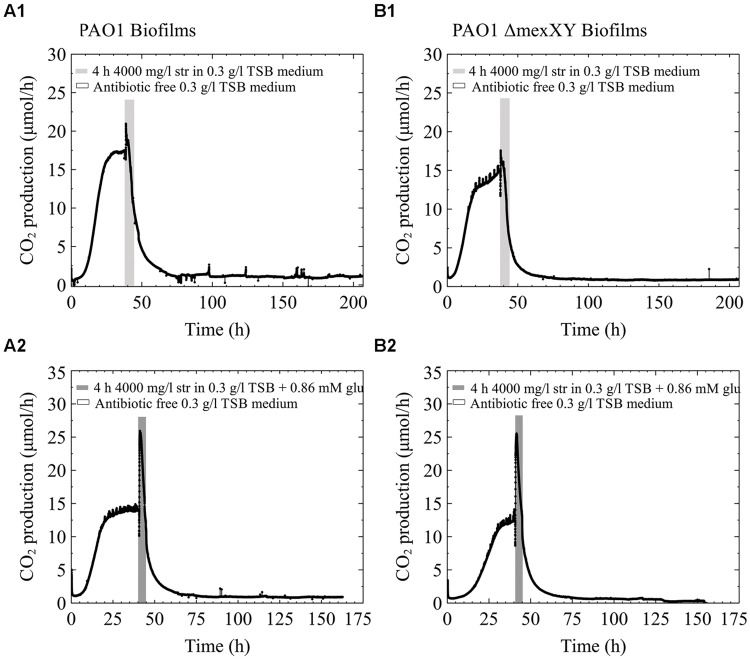
**CO_2_ production (μmol/h) of biofilms of *P. aeruginosa* PAO1 **(A)** and PAO1 ΔMexXY **(B)** grown in 0.3 g/l TSB.** The biofilms were grown for less than 48 h and had reached steady state CO_2_ output before they were exposed to 4000 mg/l streptomycin for 4 h. **(A1)** and **(B1)** biofilms were exposed to streptomycin in 0.3 g/l TSB medium. **(A2)** and **(B2)** were exposed to streptomycin in 0.3 g/l TSB medium with the addition of 0.86 mM glucose (glu).

The eﬄux pump MexXY is a known mechanism of increased resistance to aminoglycoside antibiotics and requires energy to function (Zhao et al., 1998; Webber and Piddock, 2003). For that reason we were interested to determine how a single species biofilm of PAO1 would behave in comparison to a PAO1 ΔMexXY mutant. We hypothesized that if the MexXY eﬄux pump in our multispecies biofilms containing *P. aeruginosa* played a role in the increased resistance to streptomycin antibiotics and was aided when extra carbon was added to the medium, that early steady state biofilms of the ΔMexXY mutant would not be aided by additional carbon in the antibiotic medium. Alternatively, we hypothesized that PAO1 would be aided by additional carbon due to the ability of the biofilm to utilize the excess energy toward antibiotic eﬄux. Our results demonstrate that early steady state PAO1 biofilms were not able to recover from the high dose streptomycin exposure with and without the addition of glucose to the antibiotic medium (**Figures 5A1,A2**). Similarly, the PAO1 ΔMexXY mutant exposed to 4000 mg/l of streptomycin with and without added carbon was unable to recover from the antibiotic exposure (**Figures 5B1,B2**). Each of the biofilms, regardless of whether they had the MexXY eﬄux pump or not, had an initial increase in CO_2_ production above steady state levels prior to a rapid decrease in CO_2_ output toward baseline levels, and when additional carbon was added to the antibiotic medium, the initial increase in CO_2_ production upon antibiotic exposure was greater (**Figure 5**).

**FIGURE 6:**
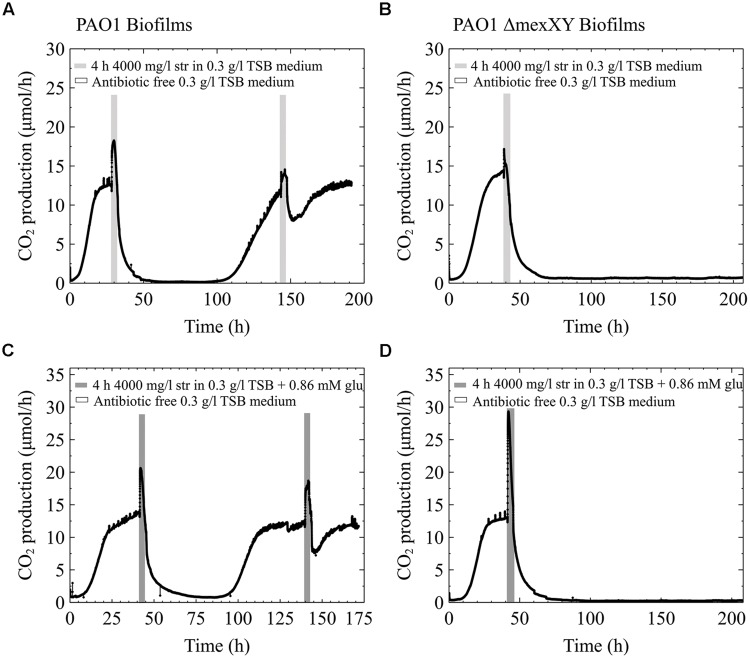
**Effect of inoculum – the experiments shown in **Figure 5** were repeated, with the only exception that the cultures used for inoculation were first streak plated prior to growing overnight cultures.** All other information as described in **Figure 5**.

Conversely, when the pure culture PAO1 freezer cultures were streak plated prior to growing overnight cultures for biofilm inoculation, the behavior differed from what is shown in **Figures 5A1,A2**. When early steady state biofilms of PAO1 originated from streak plates, as old as 2 weeks, they were able to recover from the high dose streptomycin exposure with and without the addition of carbon to the antibiotic medium (**Figures 6A1,A2**). Unlike PAO1, the PAO1 ΔMexXY strain was unable to recover from high dose streptomycin exposures even when the freezer culture was inoculated onto agar plates prior to growing overnight cultures used for biofilm inoculation (**Figures 6B1,B2**).

### Adaptation and Comparison to other Gram-Negative Strains

**FIGURE 7:**
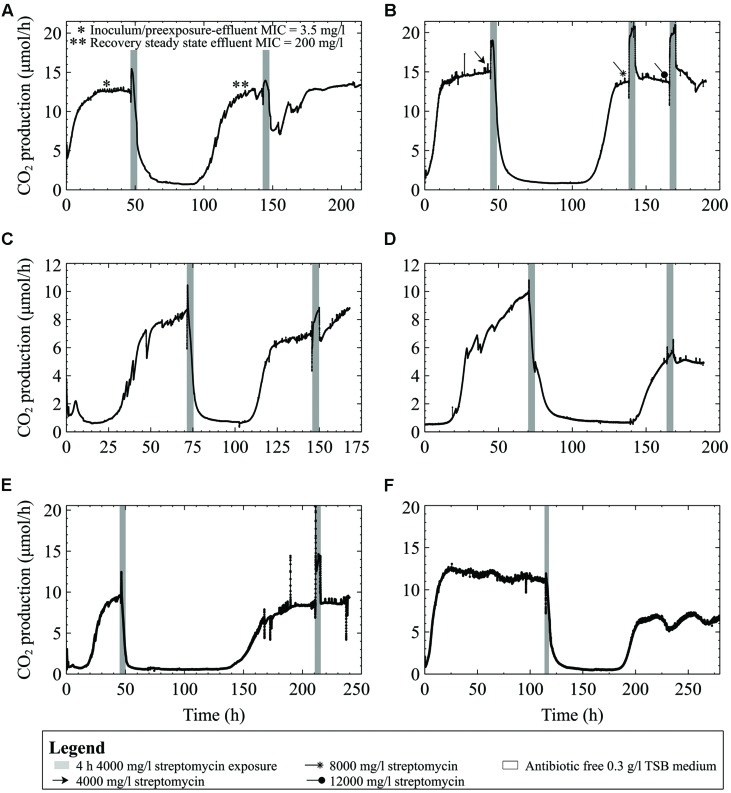
**CO_2_ production (μmol/h) of biofilms inoculated from bench cultures exposed to streptomycin in 0.3 g/l TSB medium for 4-h intervals. (A)** Multispecies culture grown on 0.3 g/l TSB medium and exposed to 4000 mg/l streptomycin. The eﬄuent cell numbers decreased from 2.0 × 10^7^ CFU/ml at steady state to 9.0 × 10^2^ CFU/ml at the end of the first 4 h exposure. In contrast, the second exposure did not result in a similar decrease of eﬄuent cell numbers; which were 2.4 × 10^5^ CFU/ml at the end of the 4 h exposure **(B)** *P. aeruginosa* on 0.3 g/l TSB medium and exposed to either 4000, 8000, or 12,000 mg/l streptomycin **(C)** an *Enterobacter* species biofilm **(D)** *Enterobacter asburiae* biofilm, and **(E)** a *Pseudomonas putida* biofilm, and **(F)** a multispecies biofilm grown for 5 days prior to the streptomycin exposure.

A culture originating from the same freezer stock culture used in the preceding experiments, but repeatedly subcultured for several years, showed the ability to recover after the first treatment at 4000 mg/l (∼600x planktonic MIC under similar growth conditions; **Figure 7A**) and without a drop in metabolism following subsequent exposures, even when the streptomycin concentration was increased to 12000 mg/l (**Figure 7B**). The numbers of viable cells released from the biofilms into the eﬄuent reflected the biofilms’ response to antibiotic exposure: as the biofilm CO_2_ production decreased, so did the number of viable cells in the eﬄuent. For example, the eﬄuent cell numbers decreased from 2.0 × 10^7^ at steady state to 1.6 × 10^3^ and 9.0 × 10^2^ CFU/ml in the eﬄuent mid exposure, and at the end of the first 4 h, respectively. In contrast, the second exposure did not result in a similar decrease in eﬄuent cell number; eﬄuent cell numbers were 2.4 × 10^5^ CFU/ml at the end of the 4 h exposure.

Tests were performed on the three Gram-negative environmental isolates to demonstrate that the effects of high doses of streptomycin on biofilm metabolism were not unique to our multispecies biofilms. Biofilms formed by the three environmental strains showed similar responses when exposed to 4000 mg/l streptomycin in low concentration (0.3 g/l) TSB medium. In each case, metabolic activity (as indicated by CO_2_ production) dropped to near-baseline levels, and took between 48 and 120 h to recover to pre-exposure levels (**Figures 7C–E**). When subsequently exposed to the antibiotic, the biofilms consistently increased their metabolism as an immediate response to the exposure, typically restoring their steady state metabolic rate to pre-exposure levels within 24 h. When a 5 days old multispecies-biofilm (inoculated from freezer stocks) was exposed to 4000 mg/l streptomycin the metabolic response resembled that of the environmental isolates and bench grown cultures (**Figure 7F**). Unlike the early steady multispecies biofilms (**Figures 2A1,A3**) the older biofilm could recover following high dose streptomycin exposures.

### Minimum Inhibitory Concentration of Streptomycin in Various Growth Medium Types

The MICs of multispecies cultures as well as the environmental isolates in low and high TSB growth medium (0.3 g/l and 3 g/l respectively) ranged between 0.5 and 3.5 mg/l. When grown in defined medium with either high or low carbon content (1 mM or 0.14 mM citrate or glucose, and 2 mM or 0.28 mM pyruvate) the multispecies culture inoculum was able to grow in higher streptomycin concentrations (from 14 mg/l up to 50 mg/l) compared to when grown in 3 g/l TSB growth medium. Previous exposure to streptomycin had an impact on the MIC of the cells released from biofilms. For instance, the streptomycin MIC of the inoculum as well as eﬄuent cells collected from biofilms grown on 0.3 g/l TSB was 3.5 mg/l, compared to eﬄuent cells collected following high dose streptomycin exposure, which had an MIC of 200 mg/l (see asterisks in **Figure 7A**).

## Discussion

Our data demonstrate that biofilm response to antibiotic exposure is dependent upon the type of medium in which the biofilm is grown, carbon availability during antibiotic exposure, the carbon source, and the length of time the biofilm is at steady state prior to exposure. In addition to nutrients and growth stage other environmental factors such as temperature, pH, or oxygen limitation will all affect biofilm response to antibiotics. In this study, the experiments were performed at 25°C that is relevant to the environmental strains used (Kropinski et al., 1987; Denton and Kerr, 1998; Hart, 2006). However, future work should include similar experiments performed under physiologically relevant temperatures of 37°C to compliment the fundamental responses of bacterial biofilms that were the main interest of this study. Mechanisms responsible for the survival of bacterial biofilms were not tested in this study, and are the focus of an ongoing investigation. Various forms of resistance have been reported in the literature, such as stable resistance and adaptive resistance that are characterized by genetic changes and down regulation of antibiotic uptake, respectively (Fernández and Hancock, 2012). Another way bacteria can survive an antibiotic exposure is through antibiotic tolerance. Antibiotic tolerance occurs when bacteria survive an antibiotic exposure through inactivity or dormancy rather than from genetic changes or active resistance mechanisms (Lewis, 2007). Under these conditions, antibiotics that normally target some form of macromolecular synthesis have no targets to act on since dormant cells are not growing and are mostly inactive (Rosenkranz, 1964; Lewis, 2007; Cotsonas King and Wu, 2009). For the purpose of this discussion the term antibiotic resistance is not used to describe biofilm behavioral changes in response to streptomycin. It has been pointed out that cells within biofilms may not have more intrinsic resistance compared to their planktonic counter parts (Lewis, 2001). We cannot speak to the growth state of individual cells from this study as we monitored whole-biofilm response. Nevertheless, the data presented here show that carbon content and growth conditions can affect microbial metabolic response to antimicrobials. Using the CEMS for monitoring whole biofilm metabolism was useful to guide experimental design for further testing to delineate antibiotic resistance mechanisms by providing cues for plausible mechanisms of tolerance and resistance. Moreover, the CEMS may provide a better simulation of real-world biofilms that typically show spatial variability.

Pre steady state and early steady state multispecies biofilms were susceptible to high concentration streptomycin exposures (**Figures 2A1,A3**), especially those younger than 48 h. This is in contrast to experiments that we have performed (**Figure 7F**) in which 4–5 days old biofilms were less susceptible to streptomycin and recovered from the same 4000 mg/l streptomycin exposure in 0.3 g/l TSB medium. This suggests that biofilm growth stage or age plays a role in biofilm susceptibility to streptomycin, which is consistent with literature (Ito et al., 2009; Chopra et al., 2015). The underlying reasons for increased survival of aged biofilms may include increased thickness, population density, changes in gene expression, and inactivation of lower layers of the biofilm (Stewart, 1994, 2002), each of which can contribute to increased antibiotic resistance and may be seen on the CO_2_ profiles as a lower overall effect on whole biofilm metabolism. Increased biofilm thickness may result in higher levels of extracellular polymeric substances (EPS) being produced such as negatively charged alginate in *P. aeruginosa* that binds to positively charged aminoglycoside antibiotics (Gordon et al., 1988; Stewart and Costerton, 2001). In addition, antimicrobials take longer to diffuse through thicker biofilms allowing more time for biofilms to upregulate adaptive stress responses, including excretion of antimicrobial degrading enzymes or increasing EPS production (Szomolay et al., 2005). Adaptive stress responses, for example nutrient stressors or antibiotic stressors, can also lead to enhanced ability to survive high concentration antibiotic exposures (Stewart, 2002; de la Fuente-Núñez et al., 2013). Furthermore, as biofilms age their cell density increases and eDNA accumulates (Fux et al., 2005; Allesen-Holm et al., 2006). Extracellular DNA can bind to cations such as Mg^2+^ and Ca^2+^ that help stabilize LPS molecules in the outer membrane. The binding of eDNA to cations leads to changes in gene expression resulting in an altered outermembrane structure more resistant to cationic antimicrobial peptides and aminoglycoside antibiotics (Mulcahy et al., 2008). Ionic binding to the outer membrane is a crucial stage in aminoglycoside uptake (Hancock, 1981).

### An Alternative Behavior for Antibiotic Survival: Increased Energy Output

Most antibiotics are effective against bacteria during their growth phase since antibiotics affect metabolic processes such as protein synthesis (aminoglycosides), DNA synthesis (fluoroquinolones), and cell wall synthesis (β-lactams; Hancock, 1981; Eng et al., 1991; Hooper, 2001; Cotsonas King and Wu, 2009). Accordingly, many studies have demonstrated the loss of antibiotic efficacy when cells are in a non-growing or starved state (Nguyen et al., 2011) and that the addition of certain nutrients can re-establish bacterial susceptibility to antibiotics (Borriello et al., 2006; Allison et al., 2011).

Contrasting the notion that the addition of nutrients increases antimicrobial susceptibility, our data demonstrate that excess nutrients provided along with the antibiotic media aided young biofilms in recovering from antibiotic exposures. Our initial hypothesis was that additional nutrients would aid biofilm recovery from aminoglycoside addition since an initial spike in metabolism upon antibiotic addition was observed. The results presented here demonstrate that the addition of excess carbon to the antibiotic medium in the form of TSB, glucose, or pyruvate reduced the young biofilms’ susceptibility to streptomycin (**Figures 2B1,B3**) compared to when no additional nutrients were added (**Figures 2A1,A3**). The spike in metabolism that occurs in the presence of the added-carbon antibiotic media typically occurs in about 15 min compared to an increase in CO_2_ output of 1 h at similar temperature as a result of bacterial doubling time (in 3 g/l TSB medium; Ronan, 2011). The large and rapid increase in metabolism (specifically **Figures 2B1** and **1B2**) when TSB or pyruvate was added to the antibiotic medium suggests that the biofilms were able to utilize the excess nutrients upon exposure, circumventing the detrimental effects of the antibiotic. Further testing would need to be performed to test the hypothesis that pyruvate or the nutrients in TSB can be utilized during streptomycin uptake. One way to test nutrient utilization would be to monitor the consumption of glucose and pyruvate before, during, and after antibiotic exposure using enzymatic assay kits. Studying how these nutrients impede negative effects of high doses of streptomycin on young biofilms may be more challenging. One would have to learn what physiological changes occur during the uptake of the carbon or nutrients and how these changes are affecting streptomycin uptake. A study performed by Buchholz et al. (2010) monitored biofilm heat-production in real-time and found that heat production spiked initially when biofilms were exposed to antibiotics, before declining along with ATP levels. Their work supports our hypothesis that metabolism increases during the antibiotic exposure and that excess nutrients can be utilized.

Previous work has shown a correlation between carbon addition and changes in antibiotic susceptibility. For example, studies performed on *E. coli*, whose preferred carbon source is glucose, have demonstrated that additional glucose leads to catabolite repression through repressing cAMP (Dalhoff, 1979) and that glucose addition can impede the antimicrobial effects of streptomycin (Zuroff et al., 2010). Furthermore, the addition of glucose in the antibiotic medium can decrease susceptibility to kanamycin in biofilms of *E. coli*; though no mechanism was given, the authors alluded to the ability of glucose to repress the uptake of other catabolites leading to antibiotic tolerance (Palmer et al., 2007). A link to nutrient uptake or metabolism and decreased susceptibility to antibiotics in *P. aeruginosa* is a possibility as well; however, the mechanisms likely differ from those in *E. coli* since even though *P. aeruginosa* does have catabolite repression control, glucose is not the preferred carbon source, but rather amino acids and organic acids (Ng and Dawes, 1967; Mukkada et al., 1973; Collier et al., 1996). The behavior of the biofilm will also depend on how the carbon sources fed into the biofilm affect other species present. We have not come across studies that have considered the behavior of *Stenotrophomonas* sp with aminoglycosides in the presence of various carbon sources. Presumably, with the limited nutrient profile of *S. maltophilia* (Stanier et al., 1966) its behavior in the presence of various nutrients would largely depend on if it could metabolize the given nutrient and/or how the other species present behave.

Only carbon sources that were readily metabolized by the biofilm enhanced survival to streptomycin; as seen when glucose, pyruvate, or TSB were added (but not citrate) to the antibiotic medium (**Figure 2A4**; **Figures 4A,D**). The inability of excess citrate in the medium to enhance survival might be explained by the fact that the TSB grown biofilms were not adapted for citrate utilization (**Figure 1A4**). TSB is a rich medium that contains glucose as the carbon source. Therefore citrate’s inability to rescue a TSB grown biofilm can potentially be explained by catabolite repression, which may occur when glucose and citrate are in the same medium. **Figures 4A–D** demonstrates the concept behind catabolite repression as the biofilms shut down their metabolism when a different carbon source was supplied in the medium. It has been shown that citrate-grown *P. aeruginosa* did not up-regulate glucose metabolizing enzymes until a threshold glucose concentration was reached, yet in glucose medium, citrate addition immediately resulted in the induction of the citrate transport system (Whiting et al., 1976). In this study the glucose and citrate were not administered at the same time thus the metabolism reflects the ability of the biofilm to utilize the carbon source administered at the time. Conversely, with addition of pyruvate to the TSB medium, bacteria can be expected to grow faster than with either a media containing glucose or pyruvate alone (Ng and Dawes, 1973).

In addition to the effects of added carbon during streptomycin exposure, the data demonstrate that the type of growth medium affects whole-biofilm metabolic response to streptomycin. Replicate experiments showed that when biofilms were grown in a defined growth medium with low (0.14 mM) carbon concentration (**Figure 3A**), biofilm susceptibility to streptomycin was diminished compared to when grown in 0.3 g/l TSB medium (containing 0.14 mM glucose). Furthermore, addition of carbon to the antibiotic medium reduced the biofilm’s susceptibility to streptomycin (**Figure 3B**). It is plausible that changes in medium composition and carbon content alter the relative abundance of the members in multispecies biofilms and their physiological responses to the antibiotic exposures.

Results of this study indicate that, in addition to the growth stage of biofilms, the origin of the culture plays a role in the metabolic recovery following a streptomycin exposure (**Figures 7A,B**). Repeated sub-culturing on agar plates might result in differential gene expression and can select for mutants best adapted for growth under laboratory conditions. It is known that decreased sensitivity to antibiotics may develop at high population density and under stressful environmental conditions that lead to rapid up-regulation of stress response genes (Mah and O’Toole, 2001). Bench cultures showed adaptation to the antibiotic by increasing their metabolic output throughout the 4-h exposures once preconditioned to the antibiotic. The increased CO_2_ output indicates that biofilms can actively prevent the antibiotic from disrupting the overall biofilm metabolism. Following a second streptomycin exposure the biofilm metabolism rapidly returned to steady state levels following the resumption of antibiotic-free growth medium.

Gram-negative environmental isolates had similar CO_2_ profiles when exposed to high concentrations of streptomycin in low concentration (0.3 g/l) TSB. Changes in the biofilm metabolic profile of the bacterial isolates, from the first antibiotic exposure to the second, demonstrate similar patterns in metabolic behavior (**Figures 7C–E**). Upon analysis of each of the biofilms’ metabolic responses to the antibiotic exposure, it was clear that the biofilms respond initially to high concentrations of streptomycin by increasing their energy output, and if primed for the exposure they will maintain their high metabolic output levels throughout the exposure. Biofilms formed by all of the environmental isolates and the lab cultures in this study were able to survive antibiotic concentrations that were higher than their planktonic MIC. The MIC’s for *Enterobacter* isolates have been reported from 8 mg/l to as high as 256 mg/l (Chiew et al., 1998); one study found streptomycin MIC of environmental strains of *P. putida* to be >512 mg/l but considered levels >16 mg/l to be the resistance breakpoint for streptomycin (Bezanson et al., 2008); environmental isolates of *S. maltophilia* showed streptomycin MIC’s from 64 mg/l even without any resistance genes detected (Popowska et al., 2012); finally, another study demonstrated the streptomycin MIC for *P. aeruginosa* was 64 mg/l in Mueller-Hinton Broth (Morita et al., 2001).

In an attempt to further explore a potential mechanism of antibiotic resistance and/or a reason for survival of young biofilms following the addition of excess carbon to the media, we tested single species biofilms of PAO1 and a PAO1 ΔMexXY mutant deficient in the ability to eﬄux streptomycin. Our initial hypothesis that the increased CO_2_ output may be linked to an energy induced antibiotic resistance mechanism was not supported based on the freezer culture inoculum. Our results cannot clearly indicate that the MexXY eﬄux pump plays a role in survival to the antibiotic as seen in **Figure 5**, since neither PAO1 nor PAO1 ΔMexXY strain could recover from the antibiotic exposures even with the addition of carbon to the antibiotic media. At this point it is not clear why the early steady state single species biofilm of PAO1 would not be aided by the addition of glucose to the antibiotic media, while the multispecies biofilms were. Interestingly, if we include the bench culture results from the pure PAO1 and ΔMexXY strains then it does appear that there is an advantage to the PAO1 culture with eﬄux pumps. The PAO1 strain had the ability to survive the antibiotic exposures while the PAO1 ΔMexXY strain did not (**Figure 6**). Therefore, there may be a link to the eﬄux pumps for the pure cultures in particular. As for the multispecies biofilms, there seems to be a link to energy requiring resistance mechanisms that can be initiated by added energy in the form of a readily useable carbon sources. This concept will need to be further explored in relation to the multispecies cultures since *P. aeruginosa* and *S. maltophilia* have different preference for carbon sources. The difference in carbon source preferences could explain why the freezer cultures from multispecies biofilms recovered upon glucose addition but the pure PAO1 cultures inoculated from freezer stocks did not. As previously stated, *P. aeruginosa* prefers organic acids to glucose (Ng and Dawes, 1967). Most naturally-occurring biofilms are formed by multispecies communities. The ways in which members of a multispecies community interact will determine biofilm physiology, structure, and behavior (Yang et al., 2011). Considering the growing evidence of multispecies infections, studies aimed at delineating interactions between members of biofilm communities during exposure to antibiotics, at physiological temperatures, merit recognition in future studies.

## Conflict of Interest Statement

The authors declare that the research was conducted in the absence of any commercial or financial relationships that could be construed as a potential conflict of interest.
