# Supplementary material for: Effect of carbon on whole-biofilm metabolic response to high doses of streptomycin
Source: Front Microbiol. 2015 Sep 11;6:953. doi: 10.3389/fmicb.2015.00953 (PMC4566048; doi:10.3389/fmicb.2015.00953)

**Supplemental Material**

CO_2_ production is continuously measured in the CEMS system, thereby creating thousands of data points for each plot. Several factors such as differences in lag phase (reported in Kroukamp et al., 2010), slight variations in metabolic response or recovery time (e.g., Figures 3 and 4 in the main document) complicate the presentation of averaged data in the Figures. However, the three replicates in Figure SM1 show that despite differences in the length of lag phase, this approach provides an instantaneous and accurate means to follow biofilm response. As indicated in the main document, biofilm stage rather than biofilm age may be more appropriate to test biofilm response to external factors.

**Figure SM1.** Response of triplicate early steady state multispecies biofilms grown on 0.3 g/l TSB medium exposed to 4000 mg/l streptomycin for 4 h.


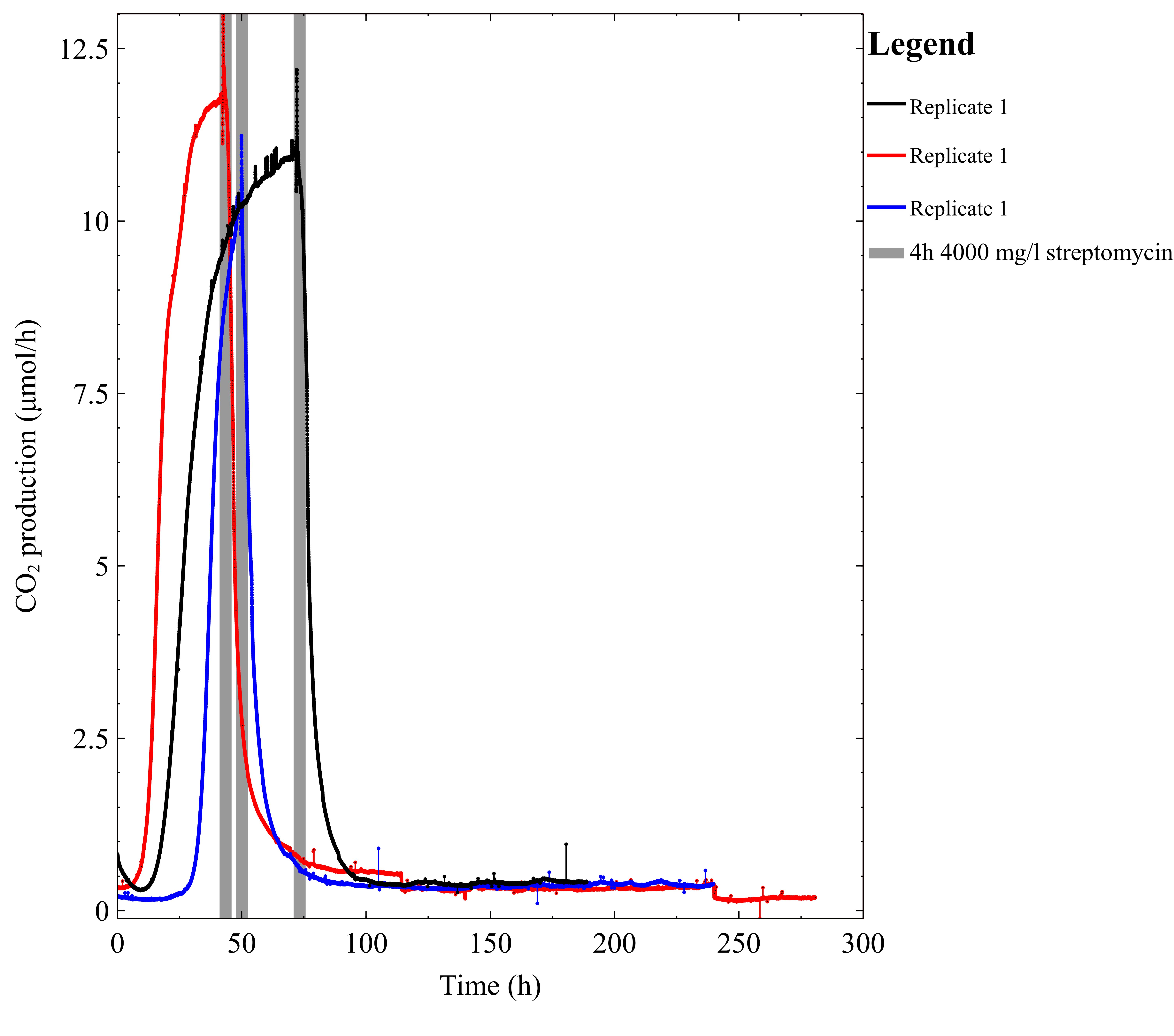

Supplement: Supplementary file 1 [file Data_Sheet_1.DOCX]
